# Pain Interference in Maintenance Hemodialysis: A Single-Center Cross-Sectional Study Using the Pain Effects Scale (PES)

**DOI:** 10.3390/jcm15031184

**Published:** 2026-02-03

**Authors:** Leszek Sułkowski, Andrzej Matyja, Maciej Matyja

**Affiliations:** 1 Department of General Surgery, Regional Specialist Hospital, Bialska 104/118, 42-218 Częstochowa, Poland; 2 Faculty of Medicine, Jan Dlugosz University in Czestochowa, Waszyngtona 4/8, 42-217 Częstochowa, Poland; 3 2nd Department of General Surgery, Medical College, Jagiellonian University, 30-688 Kraków, Poland

**Keywords:** hemodialysis, end-stage kidney disease, dialysis, Kt/V, urea reduction ratio, ultrafiltration, vascular access, kidney transplantation, pain, pain effects scale (PES)

## Abstract

**Background:** Pain is a common and clinically important symptom in hemodialysis, yet its functional impact and determinants remain insufficiently characterized. This study examined factors associated with pain interference using the Pain Effects Scale (PES) in maintenance hemodialysis patients. **Methods:** In a cross-sectional study, 73 adults receiving thrice-weekly hemodialysis completed the PES, assessing the four-week impact of pain on mood, sleep, mobility, work, recreation, and enjoyment of life. Demographic, clinical, and dialysis-related variables—including vascular access type, dialysis vintage, session duration, ultrafiltration volume, predialysis urea, Kt/V, urea reduction ratio, comorbidities, and transplant history—were extracted from medical records. Associations were evaluated using parametric and non-parametric tests. **Results:** PES scores indicated substantial pain interference. Older age was positively correlated with higher PES scores (r = 0.32, *p* = 0.006), and patients with ischemic heart disease had significantly higher PES values than those without (23.1 ± 6.7 vs. 17.3 ± 6.2; *p* = 0.012). Willingness to pursue transplantation showed a non-significant trend toward lower scores. **Conclusions:** Pain interference in hemodialysis appears largely independent of routine adequacy metrics and most comorbidities, with ischemic cardiovascular disease emerging as an exception. Findings underscore the need for a biopsychosocial approach integrating pain screening with assessment of mood, sleep, neuropathy, musculoskeletal factors, and ischemic symptoms.

## 1. Introduction

Pain is one of the most prevalent and burdensome symptoms among individuals with end-stage kidney disease treated with maintenance hemodialysis. Across observational cohorts and systematic reviews, a majority of patients report ongoing pain, often moderate to severe, which frequently co-occurs with fatigue, sleep disturbance, anxiety, and depression, and substantially erodes health-related quality of life [1,2,3,4]. Beyond discomfort, pain has clear clinical and prognostic relevance: greater pain burden is linked to poorer health-related quality of life and psychosocial distress, and contributes to the overall symptom load shaping daily functioning and well-being in hemodialysis [3,4,5,6,7]. Large multinational analyses further demonstrate that health-related quality of life domains—including bodily pain—predict hospitalization and mortality independent of conventional laboratory targets [8,9].

Symptom science in chronic kidney disease consistently shows that patients’ day-to-day well-being is shaped less by biochemical “normalization” and more by the cumulative burden of symptoms, including pain, that interfere with mobility, sleep, work or household activities, social participation, and enjoyment of life [1,4,5,6,7,10,11]. Multiple overlapping mechanisms plausibly drive pain in hemodialysis, including neuropathic processes (e.g., painful diabetic neuropathy), musculoskeletal disorders, and ischemic etiologies, which often co-occur and produce heterogeneous pain phenotypes [12,13]. Treatment-related factors also contribute: cannulation discomfort and access-site pain are well recognized by patients. Qualitative syntheses emphasize fear of needling and bodily intrusion, while comparative trials show mixed effects of specific needling techniques on pain [14,15]. Intradialytic fluid removal practices may further influence cramping, post-dialysis “hangover,” and recovery time, symptoms often experienced as painful or pain-like, particularly at higher ultrafiltration intensities [16,17,18]. Symptom intensity and interference also vary across and within dialysis days, underscoring the need for standardized, time-aware, patient-reported assessments [19].

Routine adequacy metrics (e.g., predialysis urea, urea reduction ratio, Kt/V) correlate only weakly with how patients feel and function. Multiple studies and reviews report weak and inconsistent associations between small-solute clearance within standard thrice-weekly ranges and patient-reported symptoms, including pain, and secular improvements in adequacy have not consistently translated into better health-related quality of life [3,7,20]. When dialysis delivery is intensified, some health-related quality of life domains may improve and post-dialysis recovery may shorten, but benefits for pain remain heterogeneous [9,16,21,22,23,24,25]. In contrast, patient-centered factors—comorbidity burden, mood and sleep disturbance, neuropathic and musculoskeletal mechanisms, and social context—often track more closely with pain and its consequences [1,4,10].

Because pain is ultimately a subjective, lived experience, centering patients’ perceptions is essential. Patient-reported outcome measures make symptoms visible to clinicians and researchers, facilitate shared decision-making, and guide targeted, multimodal care in dialysis units [8]. Importantly, patients distinguish pain intensity from pain interference—that is, the extent to which pain disrupts mood, mobility, sleep, work and household roles, recreation, and enjoyment of life—domains they identify as meaningful [25,26]. The Pain Effects Scale (PES) was developed to capture pain interference succinctly across six items, is easy to self-administer, and has strong psychometric support from the Medical Outcomes Study framework and related instruments [27,28,29]. By focusing on interference over the preceding four weeks, the PES complements dialysis-specific instruments and facilitates translation of symptom data into patient-centered care [25].

Despite growing attention to patient-reported outcomes in nephrology, determinants of pain interference, as distinct from pain intensity, remain incompletely characterized in hemodialysis. Associations with demographic factors, comorbid conditions, vascular access type, and treatment parameters have been heterogeneous or modest across studies. Recent multinational work suggests that psychosocial and clinical context often outweighs dialysis dose in explaining pain interference [8,9,10,16,21,30,31,32,33,34]. Social resources also appear influential, as perceived support from family and friends correlates more robustly with patient-reported outcomes than administrative markers such as marital status [35]. Parallel research documents substantial burdens of fatigue, cognitive, sexual, and visual limitations and other social determinants among hemodialysis patients—domains that cluster with pain and may amplify its impact on daily life [10,11,36,37,38,39,40,41,42].

Kidney transplantation remains the preferred therapy for eligible patients and is associated, on average, with better patient-reported outcomes than maintenance dialysis. However, clinically meaningful pain often persists after transplantation, and decisional factors such as knowledge, attitudes, and systemic barriers may shape willingness to pursue transplantation independent of symptom severity [6,43,44,45]. Understanding how pain interference relates to transplant history and readiness has practical implications for counseling and care planning within dialysis programs.

In this cross-sectional study, we used the PES questionnaire to characterize the distribution and magnitude of pain interference in a single-center cohort of maintenance hemodialysis patients; examine associations between PES scores and sociodemographic characteristics, comorbidities, and dialysis-related features; and explore whether transplant history and willingness to undergo transplantation are linked to differences in pain interference. By focusing on pain interference rather than intensity and by evaluating its relationship not only with clinical and dialysis parameters but also with transplant-related attitudes, this study addresses an underexplored dimension of symptom burden in hemodialysis. Situating PES alongside routinely collected clinical metrics aims to highlight discrepancies between biochemical adequacy and lived symptom burden and to address a gap in understanding the functional impact of pain in this population.

## 2. Materials and Methods

We conducted a cross-sectional study in the Dialysis Unit of the Regional Specialist Hospital in Częstochowa, Poland. The research protocol received approval from the local Bioethics Committee (K.B.Cz. 0014/2017), and all procedures were performed in accordance with the Declaration of Helsinki. Before enrollment, all participants were provided with written information regarding study objectives and procedures, and subsequently gave written informed consent. Eligible patients were adults (≥18 years) with end-stage kidney disease undergoing maintenance hemodialysis. Exclusion criteria included acute kidney injury (a transient, unstable clinical state associated with fluctuating symptoms), current peritoneal dialysis (a modality with distinct symptom patterns and treatment characteristics that would introduce clinical heterogeneity), age < 18 years, refusal to participate, incomplete questionnaire data, and clinically significant cognitive impairment precluding reliable self-report. During the study period, 135 patients were receiving maintenance hemodialysis in our unit. Of these, 62 were excluded based on predefined criteria, and the remaining 73 consecutive eligible patients formed the final sample included in the analysis. Reasons for refusal or non-participation were not systematically collected.

Questionnaires were administered during mid-week dialysis sessions (Wednesday or Thursday) to minimize variability related to intradialytic timing and clinical stability. Patients completed the survey within the unit and were not permitted to take it home to ensure standardized conditions. Participants completed the forms independently, without family members or clinical staff present. A trained interviewer was available only to clarify item wording if needed. All responses referred to the preceding four weeks.

To quantify the perceived impact of pain on everyday functioning, we used the PES, a six-item self-report instrument originating from the Medical Outcomes Study [27,28,29]. Because no validated Polish-language version of the PES was available at the time of the study, we prepared a Polish translation of the instrument. Using five-point Likert scales (1 = not at all; 5 = extremely), respondents rated the degree to which pain or unpleasant bodily sensations over the past four weeks interfered with mood, walking or general mobility, sleep, work or household activities, recreation or leisure, and quality of life. Total scores range from 6 to 30, with higher scores reflecting a greater adverse impact of pain [27,28,29]. The PES is designed for self-administration and is typically well understood without interviewer assistance. The PES assesses patient-reported pain interference with daily functioning, independent of pain etiology. Pain phenotypes were not differentiated, as the study focused on patients’ subjective perception of pain impact rather than clinician-defined mechanisms. In accordance with the original instrument manuals, “pain” is broadly defined to include any unpleasant sensory symptoms, regardless of etiology, and scores are conventionally analyzed as continuous variables. Prior psychometric evaluations demonstrate high internal consistency and construct validity, including expected correlations with generic pain and role-function domains, and relative independence from neurological impairment severity [27,28,29].

Demographic data (age, sex, marital status, and educational attainment) and treatment characteristics were obtained from medical records and same-day patient reports. Dialysis-related variables included vascular access type (arteriovenous fistula or central venous catheter), dialysis vintage (in months), session duration (hours), ultrafiltration volume per session (mL), predialysis urea concentration (mg/dL), and adequacy indices (Kt/V and urea reduction ratio). In the study, we included comorbidities such as hypertension, diabetes mellitus, and ischemic heart disease, which are highly prevalent in hemodialysis populations, are consistently linked to pain mechanisms. Information on the history of kidney transplantation (yes/no) and willingness to pursue future transplantation (yes/no) was also collected.

### Statistical Analysis

Continuous variables were summarized as mean ± standard deviation (SD) and categorical variables as frequencies and percentages. Normality was evaluated using the Shapiro–Wilk test and visual inspection of histograms. Homogeneity of variances was assessed with Levene’s test.

Between-group comparisons for continuous variables were performed using independent-samples *t*-tests or one-way ANOVA when assumptions were met. When normality or variance assumptions were violated, non-parametric Mann–Whitney U or Kruskal–Wallis tests were applied. For two-group comparisons with unequal variances, Welch’s *t*-test was used. Effect sizes were calculated when relevant. Associations between PES scores and continuous clinical variables were examined using Pearson correlation for normally distributed variables and Spearman rank correlation otherwise. All statistical tests were two-tailed, and a *p*-value < 0.05 was considered statistically significant. No adjustments for multiple comparisons were applied due to the exploratory nature of the study. Multivariable regression analyses were not performed due to the exploratory nature of the study, the modest sample size, and small subgroup counts, which would have increased the risk of overfitting and biased adjustment. Statistical analyses were conducted using IBM SPSS Statistics version 28 (IBM Corp., Armonk, NY, USA).

## 3. Results

Seventy-three consecutive patients meeting the inclusion criteria were enrolled. PES scores ranged from 6 to 30 (mean ± SD 18.04 ± 6.51). Age ranged from 28 to 84 years (mean ± SD 61.62 ± 12.23). A statistically significant positive correlation was observed between age and PES (Pearson r = 0.320, *p* = 0.0058), indicating greater pain interference with increasing age (Table 1). However, this association is unadjusted and should be interpreted cautiously given the exploratory design.

The study population consisted of 46 males and 27 females. PES scores ranged from 6 to 30 among males and 6–29 among females. Mean PES values were comparable between the two groups (males: 18.13 ± 6.66 vs. females: 17.89 ± 6.38; *p* = 0.3425). Both distributions approximated normality, and an independent-samples *t*-test demonstrated no significant difference in PES between genders (*p* = 0.8796).

Regarding marital status, the cohort included 11 single, 51 married, and 11 widowed participants. Mean PES scores were 16.00 ± 6.18, 18.04 ± 6.59, and 20.09 ± 6.36, respectively. PES distributions were normally distributed with homogeneity of variances. A one-way ANOVA revealed no statistically significant differences in PES across marital-status categories (*p* = 0.3425).

Educational attainment included primary (*n* = 41), secondary (*n* = 20), and university-level education (*n* = 12). Mean PES scores in these groups were 18.61 ± 7.00, 17.75 ± 5.76, and 16.58 ± 6.22, respectively. One-way ANOVA indicated no significant association between education level and PES (*p* = 0.627).

Sixty-two patients used an arteriovenous fistula and 11 used a central venous catheter. Mean PES scores were 17.95 ± 6.75 and 18.55 ± 5.16, respectively. No significant difference was observed between groups (*p* = 0.7826).

Dialysis duration ranged from 1 to 317 months (mean ± SD 48.95 ± 63.05). PES scores were normally distributed, whereas dialysis duration was right-skewed. Correlation analyses showed no significant association between PES and dialysis duration (Pearson r = 0.069, *p* = 0.5635; Spearman ρ = 0.035, *p* = 0.7671).

Hypertension (n = 50) and diabetes mellitus (n = 21) were not associated with significant PES differences (hypertension *p* = 0.8465; diabetes *p* = 0.1309). In contrast, patients with ischemic heart disease (n = 9) had significantly higher PES scores compared with those without (23.11 ± 6.66 vs. 17.33 ± 6.22; *p* = 0.0116) (Table 2).

Six patients reported a history of kidney transplantation. PES scores did not differ significantly between patients with and without transplant history (17.83 ± 6.59 vs. 18.06 ± 6.55; *p* = 0.9357).

Thirty-eight patients declined and 35 expressed willingness to pursue transplantation (Table 3). Mean PES scores were 19.29 ± 5.83 and 16.69 ± 7.01, respectively. Although the difference did not reach statistical significance (*p* = 0.0879), Cohen’s d (0.41) indicated a small-to-moderate effect size favoring lower PES among patients willing to undergo transplantation.

Predialysis urea averaged 125.7 ± 30.6 mg/dL (range 72–202 mg/dL) (Table 4). No significant correlation was observed between PES and urea levels (Pearson r = 0.15, *p* = 0.194). Mean session duration was 3.93 ± 0.34 h. Neither Pearson (r = −0.188, *p* = 0.1111) nor Spearman correlation (ρ = −0.187, *p* = 0.1136) indicated significant associations with PES. Comparing sessions ≤ 4 h (n = 62) versus >4 h (n = 11), no significant difference in PES was detected (18.42 ± 6.37 vs. 15.91 ± 7.22; *p* = 0.2413). Mean ultrafiltration volume was 2256.2 ± 923.6 mL (range 300–4200 mL). Weight-normalized ultrafiltration rates were not calculated. Pearson correlation showed no significant association with PES (Pearson r = 0.10, *p* = 0.397). Kt/V values averaged 1.28 ± 0.25 (0.76–1.89). No significant correlation was observed between PES and Kt/V (Pearson r = −0.15, *p* = 0.209). Mean urea reduction ratio was 0.66 ± 0.08 (0.48–0.90). PES was not significantly correlated with urea reduction ratio (Pearson r = −0.164, *p* = 0.165).

## 4. Discussion

In this single-center cohort of maintenance hemodialysis patients, two variables—older age and the presence of ischemic heart disease—were significantly associated with higher pain interference as measured by the PES. These findings should be interpreted as hypothesis-generating rather than confirmatory, given the exploratory design, univariable analyses, and modest sample size.

The modest positive association between age and PES scores is consistent with broader chronic kidney disease literature, indicating that older adults often report greater interference of symptoms with daily functioning, even when overall symptom burden is comparable [37,46]. Foundational dialysis studies likewise document that pain is common and meaningfully impairs health-related quality of life, supporting the relevance of measuring pain interference rather than intensity alone [5,6]. However, existing evidence suggests that age is rarely a dominant determinant of pain interference once psychosocial and clinical factors—such as depression, multimorbidity, and sleep disturbance—are considered [11,13,28,30]. The small effect size observed in our cohort aligns with prior systematic reviews reporting heterogeneous and often modest associations between age and pain in hemodialysis, underscoring the need for larger, adequately powered studies [30].

A second key finding was the significantly higher PES scores among patients with ischemic heart disease. This association is biologically plausible, as ischemic cardiovascular conditions may contribute to pain through reduced peripheral perfusion, ischemic limb pain, angina, or musculoskeletal deconditioning. Observational studies demonstrate that peripheral arterial disease and ischemic cardiovascular pathology are common in dialysis populations and are associated with substantial pain interference and functional limitation [47]. Although the ischemic heart disease subgroup in our study was small, the direction and magnitude of the observed association are consistent with these mechanisms. Importantly, broader literature indicates that overall comorbidity burden often explains more variance in pain interference than individual diagnoses [30], and unmeasured factors such as depression or sleep disorders may partially confound this relationship.

In contrast, hypertension and diabetes mellitus were not significantly associated with PES scores. While diabetes has strong biological plausibility as a pain driver via peripheral and autonomic neuropathy, studies using structured neuropathic pain assessments suggest that diabetes is more strongly linked to neuropathic pain phenotypes than to global pain interference scores [12,13]. The lack of association observed here may therefore reflect limited statistical power, heterogeneity of pain mechanisms, or the fact that PES captures functional impact rather than pain type. Similarly, hypertension alone may exert limited influence on pain interference once psychosocial and comorbidity-related factors are considered [11,26].

No significant differences in PES scores were observed across sex, marital status, or educational attainment. Prior literature on demographic predictors of pain in hemodialysis is mixed, with many studies reporting attenuation or loss of significance after adjustment for clinical and psychosocial variables [1,2,10,28]. In particular, marital status appears to be a crude proxy for social resources; perceived social support has been shown to correlate more robustly with patient-reported outcomes than legal marital status [35]. Our null findings may therefore reflect limited power or the inability of simple sociodemographic markers to capture the interpersonal and psychosocial factors that modulate pain interference.

Pain interference was also not associated with vascular access type, dialysis vintage, or routine dialysis adequacy parameters, including predialysis urea, Kt/V, urea reduction ratio, session duration, and ultrafiltration volume. These results align with a substantial body of evidence showing weak and inconsistent relationships between small-solute clearance metrics and patient-reported symptoms, including pain [3,7,8,20]. Although access-related pain—particularly cannulation discomfort—is clinically meaningful and modifiable [14,15], episodic needling pain may not translate into higher global pain interference scores over several weeks, especially when other chronic pain contributors predominate. Similarly, while higher ultrafiltration rates are linked to intradialytic symptoms such as cramping and headache [3,17,18,32], PES emphasizes longer-term functional impact, which may be driven more strongly by chronic neuropathic, musculoskeletal, inflammatory, and psychosocial factors.

Regarding kidney transplantation, prior transplant history was not associated with lower PES scores, consistent with the literature showing that although transplantation improves many quality-of-life domains, clinically relevant pain often persists [43,44,45]. Persistent post-transplant pain may reflect irreversible neuropathy, osteoarticular disease, central sensitization, or treatment-related syndromes such as calcineurin-inhibitor–induced pain [12,48]. Patients expressing willingness to pursue transplantation showed a non-significant trend toward lower PES scores, with a small-to-moderate effect size. While not statistically significant, this directional signal is consistent with evidence linking higher symptom burden to poorer functioning and reduced treatment engagement [49]. At the same time, willingness to pursue transplantation is shaped by complex interactions among symptoms, knowledge, attitudes, and structural barriers, indicating that pain is only one component of transplant decision-making [14,31,45].

Taken together, our findings reinforce the concept that pain interference in hemodialysis is a multidimensional, biopsychosocial phenomenon that is only weakly related to routine dialysis adequacy metrics. The significant associations observed for age and ischemic heart disease should be viewed as exploratory signals requiring confirmation in larger, multicenter studies with comprehensive covariate adjustment. The predominance of null findings does not indicate absence of effect but likely reflects limited statistical power, heterogeneous pain mechanisms, and measurement constraints inherent to single-center cross-sectional designs.

### Limitations and Perspectives for Future Research

This study has several limitations. Its cross-sectional, single-center design limits causal inference and generalizability. The overall sample was modest, reducing power to detect small-to-moderate effects and widening confidence intervals. The findings should be interpreted as exploratory and hypothesis-generating, as all associations are unadjusted and may be influenced by confounding factors, small subgroup sizes, and limited statistical power. Pain was captured with the PES, which measures interference over the preceding four weeks. Although widely used, it is not kidney-specific, it does not distinguish pain phenotypes and may under-represent acute intradialytic symptoms. As a result, mechanistic inferences regarding neuropathic, nociceptive, or ischemic pain contributors cannot be made, and future studies should incorporate structured pain phenotyping instruments. All exposures and outcomes were self-reported (except routine clinical data), introducing potential recall and reporting biases. Detailed information on analgesic use was not systematically collected, although all patients received individualized pain management as part of routine nephrology care. Analgesic treatment patterns should be addressed in future studies. As no validated Polish-language version of the PES was available, an author-translated Polish version was used to ensure patient comprehension.

Future studies should adopt multicenter, adequately powered, and preferably longitudinal designs to assess trajectories of pain interference and enable causal modeling; incorporate comprehensive covariate panels—including mood, sleep, frailty, physical activity, inflammatory markers, and granular comorbidity indices—to clarify mechanisms and confounding; perform structured pain phenotyping; evaluate access-focused interventions to mitigate cannulation pain. Finally, studies should evaluate whether improvements in pain interference translate into better transplantation readiness.

## 5. Conclusions

In this single-center cohort of maintenance hemodialysis patients, PES-measured pain interference was modestly higher in older individuals. Ischemic heart disease was associated with significantly higher reported pain, whereas hypertension and diabetes mellitus were not related to PES scores. A history of kidney transplantation was not associated with PES, and only a non-significant trend suggested lower PES among patients willing to pursue transplantation. By contrast, PES scores did not differ by sex, marital status, or educational attainment, and showed no association with vascular access type (arteriovenous fistula vs. central venous catheter), dialysis vintage, single-session duration, ultrafiltration volume, or dialysis adequacy indices, including predialysis urea, Kt/V, and urea reduction ratio. Together, these findings indicate that the impact of pain in hemodialysis is largely decoupled from routine adequacy metrics. Given the cross-sectional design and modest sample size, these results should be interpreted as hypothesis-generating and warrant confirmation in larger cohorts.

These preliminary findings highlight the importance of further research rather than direct clinical implementation. Larger, multicenter, longitudinal studies with multivariable modeling are required to validate these observations and to clarify causal pathways and clinical relevance before drawing conclusions for clinical practice.

## Figures and Tables

**Table 1:** Association between Pain Effects Scale (PES) and demographics and sociocultural parameters, vascular access, and dialysis duration.

		*n*	PES Mean ± SD (Range)	*p*-Value
PES		73	18.04 ± 6.51 (6–30)	
Age	(years)	73	62.62 ± 12.23 (28–84)	Pearson r = 0.320; *p* = 0.0058
Gender	male	46	18.13 ± 6.66 (6–30)	0.8796
	female	27	17.89 ± 6.38 (6–29)	
Marital status	single	11	16.00 ± 6.18 (7–24)	0.3425
	married	51	18.04 ± 6.59 (6–30)	
	widowed	11	20.09 ± 6.36 (8–29)	
Education	primary	41	18.61 ± 7.00 (6–30)	0.627
	secondary	20	17.75 ± 5.76 (7–29)	
	university	12	16.58 ± 6.22 (7–24)	
Vascular access for dialysis	arterio-venous fistula	62	17.95 ± 6.75 (6–30)	0.7826
	central venous catheter	11	18.55 ± 5.16 (6–24)	
dialysis duration	(months)	73	48.95 ± 63.05 (1–317)	Pearson r = 0.069; *p* = 0.5635 Spearman ρ = 0.035, *p* = 0.7671

PES—Pain Effect Scale, SD—standard deviation.

**Table 2:** Association between Pain Effects Scale (PES) and comorbidities.

	Hypertension		Diabetes Mellitus		Ischemic Heart Disease	
	yes	no	yes	no	yes	no
*n* PES mean ± SD (range)	50 17.94 ± 6.54 (6–30)	23 18.26 ± 6.59 (7–29)	21 19.86 ± 6.66 (10–29)	52 17.31 ± 6.37 (6–30)	9 23.11 ± 6.66 (10–29)	64 17.33 ± 6.22 (6–30)
*p*	0.8465		0.1309		0.0116	

PES—Pain Effect Scale, SD—standard deviation.

**Table 3:** Association between Pain Effects Scale (PES) and kidney transplantation.

		*n*	PES Mean ± SD (Range)	*p*-Value
History of kidney transplantation	yes	6	17.83 ± 6.59 (9–26)	0.9357
	no	67	18.06 ± 6.55 (6–30)	
Willingness for kidney transplantation	yes	35	16.69 ± 7.01 (6–29)	0.0879
	no	38	19.29 ± 5.83 (6–30)	

PES—Pain Effect Scale, SD—standard deviation.

**Table 4:** Association between Pain Effects Scale (PES) and dialysis treatment parameters.

	n	Mean ± SD (Range)	Correlation with PES Score
urea (mg/dL)	73	125.7 ± 30.6 (72–202)	Pearson r = 0.15; *p* = 0.194
duration of dialysis session (hours)	73	3.93 ± 0.34 (3.0–5.0)	Pearson r = −0.188; *p* = 0.1111 Spearman ρ = −0.187, *p* = 0.1136
ultrafiltration (mL)	73	2256.2 ± 923.6 (300–4200)	Pearson r = 0.10; *p* = 0.397
Kt/V	73	1.28 ± 0.25 (0.76–1.89)	Pearson r = −0.15; *p* = 0.209
urea reduction ratio	73	0.66 ± 0.08 (0.48–0.90)	Pearson r = −0.164; *p* = 0.165

PES—Pain Effect Scale, SD—standard deviation.

## Data Availability

The data presented in this study are available on request from the corresponding author. The data are not publicly available due to privacy restrictions.

## Abbreviations

The following abbreviations are used in this manuscript:

PES	Pain Effect Scale
SD	Standard deviation

## References

[1] Murtagh F.E., Addington-Hall J., Higginson I.J. (2007). The prevalence of symptoms in end-stage renal disease: A systematic review. Adv. Chronic Kidney Dis..

[2] Brkovic T., Burilovic E., Puljak L. (2016). Prevalence and severity of pain in adult end-stage renal disease patients on chronic intermittent hemodialysis: A systematic review. Patient Prefer. Adherence.

[3] Weisbord S.D., Fried L.F., Arnold R.M., Fine M.J., Levenson D.J., Peterson R.A., Switzer G.E. (2005). Prevalence, severity, and importance of physical and emotional symptoms in chronic hemodialysis patients. J. Am. Soc. Nephrol..

[4] Davison S.N., Jhangri G.S. (2005). The impact of chronic pain on depression, sleep, and the desire to withdraw from dialysis in hemodialysis patients. J. Pain Symptom Manag..

[5] Davison S.N., Jhangri G.S., Johnson J.A. (2006). Cross-sectional validity of a modified Edmonton symptom assessment system in dialysis patients: A simple assessment of symptom burden. Kidney Int..

[6] Masajtis-Zagajewska A., Pietrasik P., Krawczyk J., Krakowska M., Jarzębski T., Pietrasiewicz B., Zbróg Z., Nowicki M. (2011). Similar prevalence but different characteristics of pain in kidney transplant recipients and chronic hemodialysis patients. Clin. Transplant..

[7] Davison S.N., Koncicki H., Brennan F. (2014). Pain in chronic kidney disease: A scoping review. Semin. Dial..

[8] Mapes D.L., Lopes A.A., Satayathum S., McCullough K.P., Goodkin D.A., Locatelli F., Fukuhara S., Young E.W., Kurokawa K., Saito A. (2003). Health-related quality of life as a predictor of mortality and hospitalization: The Dialysis Outcomes and Practice Patterns Study (DOPPS). Kidney Int..

[9] Manns B., Johnson J.A., Taub K., Mortis G., Ghali W.A., Donaldson C. (2003). Quality of life in patients treated with hemodialysis or peritoneal dialysis: What are the important determinants?. Clin. Nephrol..

[10] Clark-Cutaia M.N., Rivera E., Iroegbu C., Arneson G., Deng R., Anastasi J.K. (2022). Exploring the Evidence: Symptom Burden in Chronic Kidney Disease. Nephrol. Nurs. J..

[11] Abdel-Kader K., Unruh M.L., Weisbord S.D. (2009). Symptom burden, depression, and quality of life in chronic and end-stage kidney disease. Clin. J. Am. Soc. Nephrol..

[12] Pop-Busui R., Boulton A.J., Feldman E.L., Bril V., Freeman R., Malik R.A., Sosenko J.M., Ziegler D. (2017). Diabetic Neuropathy: A Position Statement by the American Diabetes Association. Diabetes Care.

[13] Sadigova E., Ozkurt S., Yalcin A.U. (2020). Pain Assessment in Hemodialysis Patients. Cureus.

[14] Casey J.R., Hanson C.S., Winkelmayer W.C., Craig J.C., Palmer S., Strippoli G.F., Tong A. (2014). Patients’ perspectives on hemodialysis vascular access: A systematic review of qualitative studies. Am. J. Kidney Dis..

[15] MacRae J.M., Ahmed S.B., Atkar R., Hemmelgarn B.R. (2012). A randomized trial comparing buttonhole with rope ladder needling in conventional hemodialysis patients. Clin. J. Am. Soc. Nephrol..

[16] Garg A.X., Suri R.S., Eggers P., Finkelstein F.O., Greene T., Kimmel P.L., Kliger A.S., Larive B., Lindsay R.M., Pierratos A. (2017). Frequent Hemodialysis Network Trial Investigators. Patients receiving frequent hemodialysis have better health-related quality of life compared to patients receiving conventional hemodialysis. Kidney Int..

[17] Churchill D.N. (1996). Sodium and water profiling in chronic uraemia. Nephrol. Dial. Transplant..

[18] Mann H., Stiller S. (2000). Sodium modeling. Kidney Int. Suppl..

[19] Kallem C.J., Alghwiri A.A., Yabes J., Erickson S., Han Z., Roumelioti M.E., Steel J.L., Jhamb M., Unruh M. (2024). Diurnal and Daily Symptom Variation in Patients with End Stage Kidney Disease: An Ecological Momentary Assessment Study. Clin. J. Am. Soc. Nephrol..

[20] Gabbay E., Meyer K.B., Griffith J.L., Richardson M.M., Miskulin D.C. (2010). Temporal trends in health-related quality of life among hemodialysis patients in the United States. Clin. J. Am. Soc. Nephrol..

[21] Hall Y.N., Larive B., Painter P., Kaysen G.A., Lindsay R.M., Nissenson A.R., Unruh M.L., Rocco M.V., Chertow G.M. (2012). Frequent Hemodialysis Network Trial Group. Effects of six versus three times per week hemodialysis on physical performance, health, and functioning: Frequent Hemodialysis Network (FHN) randomized trials. Clin. J. Am. Soc. Nephrol..

[22] Rocco M.V. (2011). Nocturnal home hemodialysis: Which of your patients should choose this modality?. Contrib. Nephrol..

[23] Culleton B.F., Walsh M., Klarenbach S.W., Mortis G., Scott-Douglas N., Quinn R.R., Tonelli M., Donnelly S., Friedrich M.G., Kumar A. (2007). Effect of frequent nocturnal hemodialysis vs conventional hemodialysis on left ventricular mass and quality of life: A randomized controlled trial. JAMA.

[24] Saran R., Bragg-Gresham J.L., Rayner H.C., Goodkin D.A., Keen M.L., Van Dijk P.C., Kurokawa K., Piera L., Saito A., Fukuhara S. (2003). Nonadherence in hemodialysis: Associations with mortality, hospitalization, and practice patterns in the DOPPS. Kidney Int..

[25] Hays R.D., Kallich J.D., Mapes D.L., Coons S.J., Carter W.B. (1994). Development of the kidney disease quality of life (KDQOL) instrument. Qual. Life Res..

[26] Lockwood M.B., Steel J.L., Doorenbos A.Z., Contreras B.N., Fischer M.J. (2021). Emerging Patient-Centered Concepts in Pain Among Adults With Chronic Kidney Disease, Maintenance Dialysis, and Kidney Transplant. Semin. Nephrol..

[27] Ritvo P.G., Fischer J.S., Miller D.M., Andrews H., Paty D.W., LaRocca N.G. (1997). Multiple Sclerosis Quality of Life Inventory: A User’s Manual.

[28] Stewart A.L., Ware J.E. (1992). Measuring Functioning and Well-Being: The Medical Outcomes Study Approach.

[29] Ware J.E., Sherbourne C.D. (1992). The MOS 36-item short-form health survey (SF-36). I. Conceptual framework and item selection. Med. Care.

[30] Brkovic T., Burilovic E., Puljak L. (2018). Risk Factors Associated with Pain on Chronic Intermittent Hemodialysis: A Systematic Review. Pain Pract..

[31] Wasse H., Kutner N., Zhang R., Huang Y. (2007). Association of initial hemodialysis vascular access with patient-reported health status and quality of life. Clin. J. Am. Soc. Nephrol..

[32] Flythe J.E., Kimmel S.E., Brunelli S.M. (2011). Rapid fluid removal during dialysis is associated with cardiovascular morbidity and mortality. Kidney Int..

[33] Jaber M.M., Abdalla M.A., Mizher A., Hammoudi H., Hamed F., Sholi A., AbuTaha A., Hassan M., Taha S., Koni A.A. (2024). Prevalence and factors associated with the correlation between malnutrition and pain in hemodialysis patients. Sci. Rep..

[34] Marzouq M.K., Samoudi A.F., Samara A., Zyoud S.H., Al-Jabi S.W. (2021). Exploring factors associated with pain in hemodialysis patients: A multicenter cross-sectional study from Palestine. BMC Nephrol..

[35] Theodoritsi A., Aravantinou M.E., Gravani V., Bourtsi E., Vasilopoulou C., Theofilou P., Polikandrioti M. (2016). Factors Associated with the Social Support of Hemodialysis Patients. Iran J. Public Health.

[36] Sułkowski L., Matyja A., Matyja M. (2025). Fatigue in Hemodialysis Patients: A Comparative Analysis with Healthy Controls. Eur. J. Investig. Health Psychol. Educ..

[37] Sułkowski L., Matyja A., Matyja M. (2025). The Role of Age in Shaping Cognitive, Physical, and Psychosocial Outcomes in Hemodialysis Patients: A Cross-Sectional Study. Medicina.

[38] Sułkowski L., Rubinkiewicz M., Matyja A., Matyja M. (2023). Visual Impairment in Hemodialyzed Patients-An IVIS Study. Medicina.

[39] Sułkowski L., Matyja A., Matyja M. (2024). Social Support and Quality of Life in Hemodialysis Patients: A Comparative Study with Healthy Controls. Medicina.

[40] Sułkowski L., Matyja A., Matyja M. (2025). The Impact of Dialysis Duration on Multidimensional Health Outcomes: A Cross-Sectional Study. J. Clin. Med..

[41] Koyama H., Fukuda S., Shoji T., Inaba M., Tsujimoto Y., Tabata T., Okuno S., Yamakawa T., Okada S., Okamura M. (2010). Fatigue is a predictor for cardiovascular outcomes in patients undergoing hemodialysis. Clin. J. Am. Soc. Nephrol..

[42] Sułkowski L., Matyja A., Matyja M. (2025). Determinants of Sexual Satisfaction in Hemodialysis Patients: A Cross-Sectional Analysis with Healthy Controls. Medicina.

[43] Lockwood M.B., Chlipala G.E., Maeinschein-Cline M., DeVon H.A., Lichvar A.B., Samra M.K., Park C.G., Campara M., Doorenbos A.Z., Tussing-Humphreys L.M. (2023). Pain Interference in End Stage Kidney Disease is Associated with Changes in Gut Microbiome Features Before and After Kidney Transplantation. Pain Manag. Nurs..

[44] Taylor K., Chu N.M., Chen X., Shi Z., Rosello E., Kunwar S., Butz P., Norman S.P., Crews D.C., Greenberg K.I. (2021). Kidney Disease Symptoms before and after Kidney Transplantation. Clin. J. Am. Soc. Nephrol..

[45] Kabbali N., Mikou S., El Bardai G., Tazi N., Ezziani M., Batta F.Z., Arrayhani M., Houssaini T.S. (2014). Attitude of hemodialysis patients toward renal transplantation: A Moroccan Interregional Survey. Transplant. Proc..

[46] Brown S.A., Tyrer F.C., Clarke A.L., Lloyd-Davies L.H., Stein A.G., Tarrant C., Burton J.O., Smith A.C. (2017). Symptom burden in patients with chronic kidney disease not requiring renal replacement therapy. Clin. Kidney J..

[47] Ašćerić R.R., Dimković N.B., Trajković G.Ž., Ristić B.S., Janković A.N., Durić P.S., Ilijevski N.S. (2019). Prevalence, clinical characteristics, and predictors of peripheral arterial disease in hemodialysis patients: A cross-sectional study. BMC Nephrol..

[48] Tsarpali V., Midtvedt K., Lønning K., Bernklev T., Lippe N.V., Reisæter A.V., Brunborg C., Heldal K. (2021). Health-Related Quality of Life in Older Kidney Transplant Recipients: A National Cohort Study of Short- and Longer-Term Outcomes. Kidney Med..

[49] Williams A., Manias E. (2008). A structured literature review of pain assessment and management of patients with chronic kidney disease. J. Clin. Nurs..

